# Biologically oriented preparation technique (BOPT) for implant-supported fixed prostheses

**DOI:** 10.4317/jced.53703

**Published:** 2017-04-01

**Authors:** Mª Fernanda Solá-Ruiz, Jaime Del Rio Highsmith, Carlos Labaig-Rueda, Rubén Agustín-Panadero

**Affiliations:** 1 DMD, PhD, MD, Adjunct Professor, Department of Oral Medicine, Faculty of Medicine and Dentistry, Valencia University, Spain; 2 DDS, Chairman of prosthodontics, Departament of Buccofacial Prosthetics, Faculty of Dentistry, Complutense, Madrid University, Spain; 3 DMD, PhD, MD, Adjunct Professor, Department of Oral Medicine, Faculty of Medicine and Dentistry, Valencia University , Spain; 4 DMD, PhD, Associate Professor, Department of Oral Medicine, Faculty of Medicine and Dentistry, Valencia University, Spain

## Abstract

A patient of 58 years of age without medical problems came to the clinic due to missing teeth in the upper posterior region and to change the partial fixed prosthesis in the upper anterior area. Proposed treatment: surgical phase of three conical shape tapering implants with prosthetic platform in occlusal direction with mechanize collar tissue level with fixtures to place implant-supported metal-ceramic restorations. In the anterior area, a zirconium oxide fixed partial prosthesis was vertical preparation of the tooth’s. When preparing teeth to receive fixed prostheses, the definition and shape of finish lines has been a subject of endless discussion, modification, and change ever since the beginnings of restorative prosthetic dentistry. The BOPT technique (biologically oriented preparation technique) was first described in the context of tooth-supported restorations but has recently been applied to dental implants with the aim of ensuring healthy peri-implant tissue and creating the possibility of modeling the peri-implant sulcus by modifying prosthetic emergence profiles. Vertical preparation of teeth and abutments without finish line on implants is a technique which was found to be adequate for ensuring the remodeling and stability of peri-implant tissues.

** Key words:**Peri-implant tissue health, shoulderless abutments.

## Introduction

Maintaining the health and stability of peri-implant soft tissues always presents a challenge in treatments with fixed implant-supported prostheses. There is a direct relationship between peri-implant mucosa health and bone tissue health. For this reason, maintaining tissue health, free of mucositis, is a guarantee of the long-term success of implant-supported prosthetic treatments (1).

The concept of vertical tooth preparation with no finishing line is applicable to fixed prostheses cemented onto implants (2,3). The use of conical implants without a finishing line makes it possible to leave a gingival margin on the prosthetic restoration (crown) rather than on the abutment, allowing the clinician to model the soft tissues and make the gingival margin level with peri-implant tissues in the same way as tooth-supported restorations. With this technique the position of the soft tissues is determined by the crown’s contours.

Aesthetics are influenced by the type of restoration and the materials involved (4).

Different factors are associated with the latter such as the patient’s gingival biotype, or iatrogenia during the implant-prosthetic treatment, implant malposition, chronic gingival inflammation due to lack of passive fit, incorrect design of the abutment preparation that is to receive bonded restorations, aggressive tooth brushing, or extravasation of cement into the peri-implant sulcus in bonded restorations with subgingival margins (5).

## Case Reports

**Figure 1:**
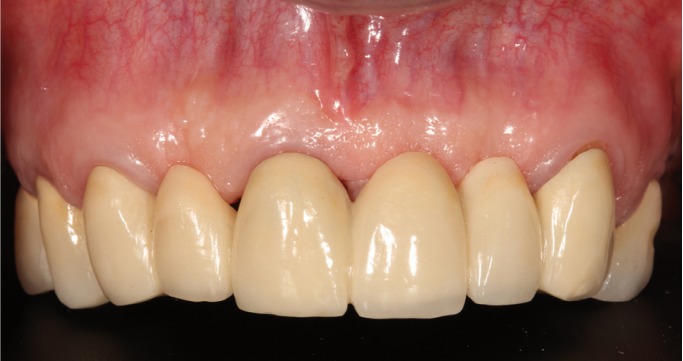
Pre-treatment image.

A patient of 58 years of age without medical problems came to the clinic due to missing teeth in the upper posterior region and to change the partial fixed prosthesis in the upper anterior area. After clinical and radiological examination, the proposed treatment was to place implant-supported metal-ceramic restorations in place of the upper right first molar, second premolar, and upper left first molar. In the anterior area, a zirconium oxide fixed partial prosthesis was planned supported by abutments on the upper right canine, upper right central incisor and upper left canine, and metal- (chromium-cobalt) ceramic one-piece restorations in place of the upper right first and second premolars and the upper left first premolar (Fig. 1).

Surgery was performed under local anesthesia (4% articaine with 1:100,000 adrenalin, Inibsa®). Three PRAMA implants (Sweden&Martina®) of 3.8 mm diameter and 10 mm length were placed supra-crestally in the positions detailed above. After surgery, oral antibiotic treatment consisted of 500 mg amoxicillin every 8 hours for 7 days (Normon®), and an anti-inflammatory, 600mg ibuprofen (Normon®) every 8 hours for 3 days.

**Figure 2:**
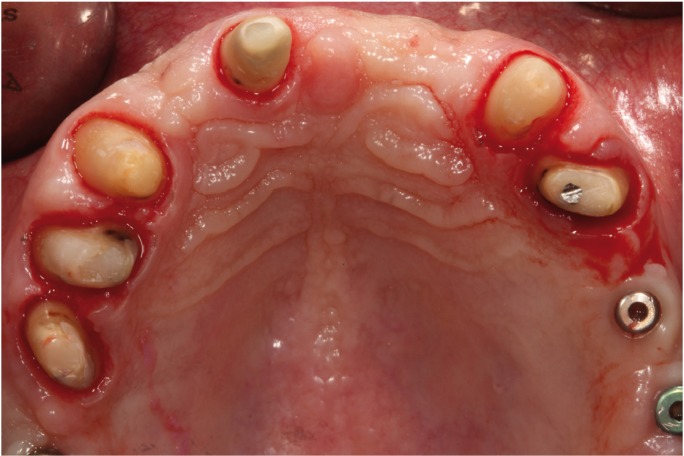
BOPT preparation of upper teeth and three implants.

At the same clinical session, the patient’s pre-existing splinted metal-ceramic fixed partial denture (from the upper right second premolar to the upper left first premolar) was removed. The teeth were prepared using the BOPT technique as described by Agustín *et al.* (3,6) (Fig. 2).

After dental preparation, a splinted acrylic resin provisional prosthesis was placed on the prepared teeth. This protocol for fabrication of interim restorations was designed to stabilize the coagulate that had formed in the gingival sulcus during the preparation. The intrasulcular zone of the interim restoration margin supported the gingival margin cir-cumferentially. The healing process determined the reinsertion and thickening of gingival tissue, which adapted to the new emergence profile (6).

**Figure 3:**
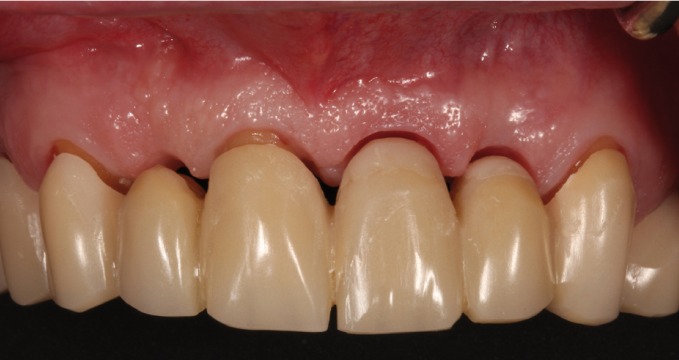
Emergence of the acrylic resin provisional prosthesis.

The interim restorations were maintained for 3 months. During this time, the prosthesis emergence was modified to achieve gingival adaptation and promote health (Fig. 3).

**Figure 4:**
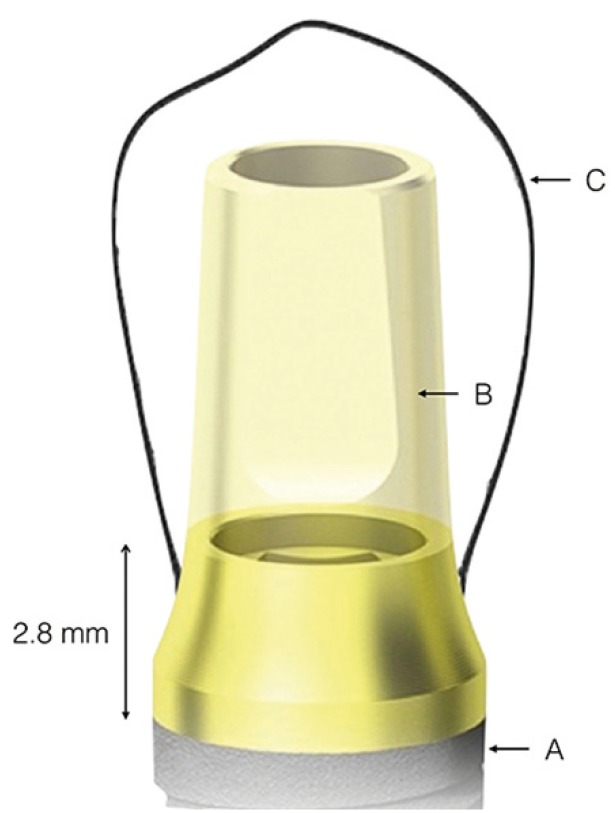
Prosthetic platform design of PRAMA implant (A), abutment (B), restoration (C).

**Figure 5:**
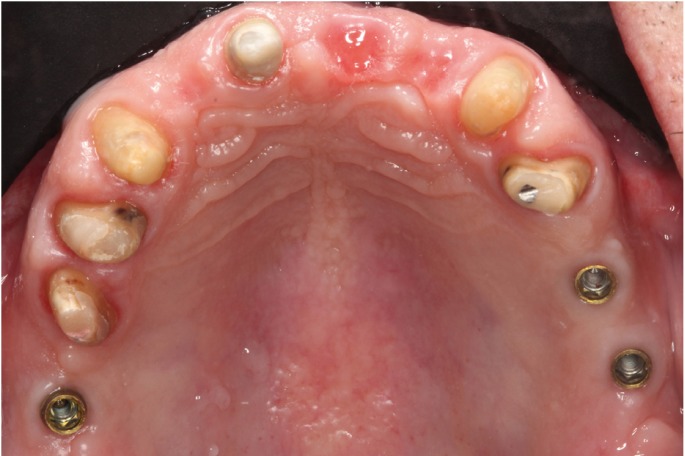
Occlusal image of keratinized gingival tissue around upper teeth and implants.

**Figure 6:**
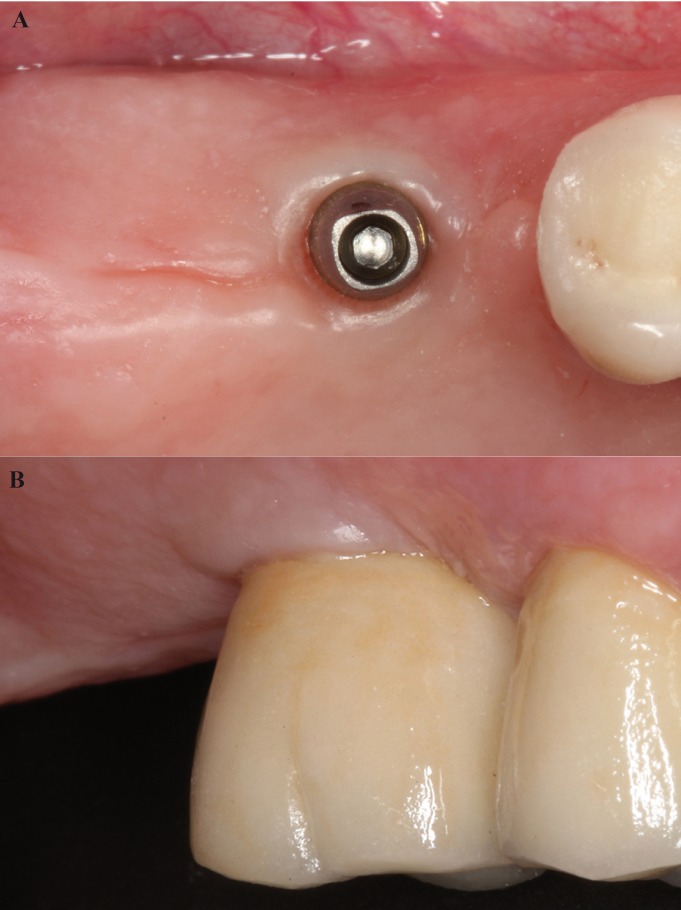
A) Occlusal image of keratinized mucosa with BOPT implant abutments in position of upper right first molar. B) Peri-implant mucosa adjacent to prosthesis in posterior area of the first quadrant 6 months after end of treatment.

**Figure 7:**
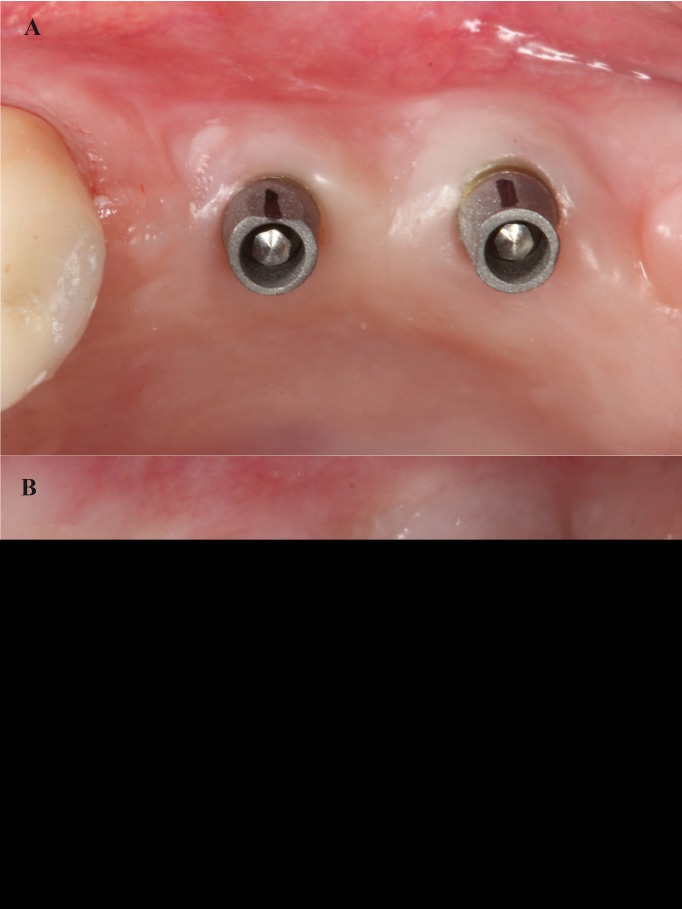
A) Occlusal image of keratinized mucosa around implants with BOPT abutments in the posterior area of the second quadrant. B) Peri-implant mucosa adjacent to prosthesis in posterior area of the second quadrant 6 months after end of treatment.

After 3 months osseointegration, prosthetic rehabilitation of the prepared implants and teeth was performed PRAMA implants (Sweden&Martina®), designed by Loi in 2015, have a shape adapted to the BOPT technique (2). The implant (A) has a machined titanium, 2,8 mm high, prosthetic platform of conical shape tapering in occlusal direction that simulates the first 2,8 mm of any prosthetic abutment without finish line. This platform (B) allows the bonded restoration to reach further in the same way as a vertical restoration would be adapted to a natural tooth, forming a vertical axial plane between implant and abutment without any horizontal gap. The prosthetic platform has an internal hexagon connection with a 3.4 mm diameter; an abutment is screwed to the platform and with a sufficient length to provide adequate retention for the restoration (C) (Fig. 4). All restorations were located 1.5 mm from the gingival margin, simulating the coronal emergence of a natural tooth above the CEJ. On tooth-supported restorations, the finish line was positioned subgingivally at 0.5 mm below the gingival margin to avoid invasion of the sulcus. (Figs. 5,6A,7A).

All restorations were fabricated using CAD/CAM technology (3Shape CAD Design Software) based on data stored in STL files obtained by extraoral scanning.

The restorations were bonded onto implants with temporary cement (Premier Implant Cement, Zeyco®), checking that no excess cement remained in the gingival groove. Restorations on teeth were cemented with dual-polymerized resin cement (RelyX Unicem 2 Automix, 3M ESPE®). The patient was instructed in oral hygiene and care of the new prostheses. Follow-up evaluations were made at three, six, twelve and 24 months after the placement of the definitive prostheses. No mechanical, esthetic or biological complications were noted.

**Figure 8:**
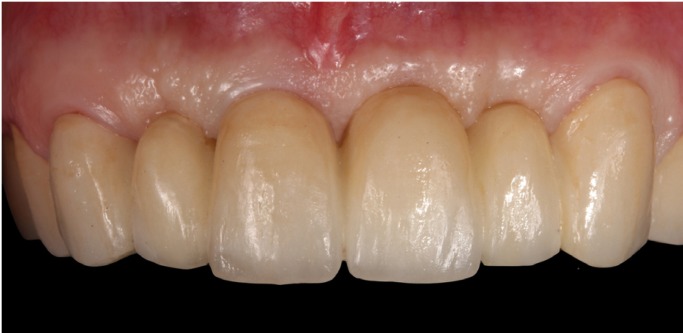
Front view post-treatment.

At the six-month follow-up, excellent peri-implant tissue health was observed, without any signs or symptoms of inflammation and with complete stability of volume and shape of the peri-implant mucosa (Figs. 6B,7B,8).

## Discussion

At present, a range of options is available to rehabilitate edentulous areas restoring both function and aesthetics. Among these, implant dentistry is becoming more popular as long-term studies have shown implant-based rehabilitation to be one of the best options, obtaining success rates of 90-95% in the medium- and long-term (7-10).

Implant dentistry has evolved rapidly in recent years due to the exponential growth in demand for this type of treatment. In this context, research into new attachments and materials is continuing apace (7).

Implant-supported restorations can be classified as screwed or bonded. Due to the angulation of the premaxilla in the anterior region, the most commonly used restorations in this area are bonded in order to overcome the difficulty presented by the angulation of the implant’s prosthetic platform.

When performing treatment with bonded restorations on implants, the principles governing design and aesthetics are the same as when bonding fixed prostheses to natural teeth (4). For this reason, biologically oriented preparation technique (BOPT), originally described in the context of tooth-supported fixed prostheses, (2,3,6) can be applied to implants in order to ensure peri-implant tissue health, without inflammation or bleeding. This also creates the opportunity to model the peri-implant sulcus by modifying the prosthetic emergence profile (4).

This implant design reduces the possible biological risks in the area of the implant-prosthetic connection resulting from the breakage of gingival desmosomal adhesion of the peri-implant tissue every time the abutment is screwed or unscrewed onto a crestally placed implant (11). In this way, the design acts less aggressively on the surrounding gingival tissue and so facilitates greater gingival stability in the medium- to long-term (12).

This would appear to be a promising treatment protocol, and could enter into common usage due to its potential to maintain the gingival tissue around prosthetic restorations. The advantages of this technique applied to implants are.

1. The possibility of repositioning the prosthetic termination line at different levels in the peri-implant sulcus. Its depth should be less than 1.5 mm as vertical over-contouring could cause invasion of the biological space and so cause peri-implant mucosa inflammation (6).

1. Optimal marginal fit due to the absence of a line supporting the restoration on the abutment. This restoration-abutment adaptation creates an area of contact that follows the concept of telescopic prosthetic design (friction between two conical surface) (3,6,13).

2. As the restoration-abutment interface simulates the cemento-enamel junction (CEJ) and emergence of a natural tooth, this allows the peri-implant mucosa to thicken and adapt to the new shape, which leads to greater gingival stability in the medium and long terms (2,3).

The drawbacks of the technique are:

1. It is a more complex technique.

2. In absence of a finish line on the abutment, it may be difficult to situate the line of the prosthetic margin correctly. When the dentist/laboratory assistant lacks experience, there is a danger of uncontrolled invasion of the sulcus.

3. The technique can only be applied to bonded restorations and may present some difficulty when it comes to removing excess bond material deriving from extravasation (6).

Vertical preparation of abutments without finish line on implants was found to be an adequate technique for ensuring the health and stability of peri-implant tissues. Medium-to long-term prospective studies are needed to confirm the clinical behavior of this type of restoration in the oral environment.
